# Mountain Pine Beetle Dynamics and Reproductive Success in Post-Fire Lodgepole and Ponderosa Pine Forests in Northeastern Utah

**DOI:** 10.1371/journal.pone.0164738

**Published:** 2016-10-26

**Authors:** Andrew P. Lerch, Jesse A. Pfammatter, Barbara J. Bentz, Kenneth F. Raffa

**Affiliations:** 1 Department of Entomology, University of Wisconsin, Madison, Wisconsin, United States of America; 2 Department of Neuroscience, University of Wisconsin, Madison, Wisconsin, United States of America; 3 United States Department of Agriculture, Forest Service, Rocky Mountain Research Station, Logan, Utah, United States of America; Natural Resources Canada, CANADA

## Abstract

Fire injury can increase tree susceptibility to some bark beetles (Curculionidae, Scolytinae), but whether wildfires can trigger outbreaks of species such as mountain pine beetle (*Dendroctonus ponderosae* Hopkins) is not well understood. We monitored 1173 lodgepole (*Pinus contorta* var. *latifolia* Doug.) and 599 ponderosa (*Pinus ponderosa* Doug. ex Law) pines for three years post-wildfire in the Uinta Mountains of northeastern Utah in an area with locally endemic mountain pine beetle. We examined how the degree and type of fire injury influenced beetle attacks, brood production, and subsequent tree mortality, and related these to beetle population changes over time. Mountain pine beetle population levels were high the first two post-fire years in lodgepole pine, and then declined. In ponderosa pine, populations declined each year after initial post-fire sampling. Compared to trees with strip or failed attacks, mass attacks occurred on trees with greater fire injury, in both species. Overall, a higher degree of damage to crowns and boles was associated with higher attack rates in ponderosa pines, but additional injury was more likely to decrease attack rates in lodgepole pines. In lodgepole pine, attacks were initially concentrated on fire-injured trees, but during subsequent years beetles attacked substantial numbers of uninjured trees. In ponderosa pine, attacks were primarily on injured trees each year, although these stands were more heavily burned and had few uninjured trees. In total, 46% of all lodgepole and 56% of ponderosa pines underwent some degree of attack. Adult brood emergence within caged bole sections decreased with increasing bole char in lodgepole pine but increased in ponderosa pine, however these relationships did not scale to whole trees. Mountain pine beetle populations in both tree species four years post-fire were substantially lower than the year after fire, and wildfire did not result in population outbreaks.

## Introduction

Most bark beetle (Coleoptera: Curculionidae, Scolytinae) species are associated with dead or stressed host trees, but some undergo intermittent population outbreaks in which they kill large numbers of apparently vigorous trees. In the past two decades bark beetle outbreaks have caused extensive tree mortality throughout western North America, affecting timber values, wildlife habitat and multiple ecosystem processes [1].

Fire-injured trees potentially provide bark beetles with a reservoir of susceptible hosts, often in a mosaic of variably injured trees across the landscape. Several species of bark beetles are known to attack fire-injured trees [2–6]. Following the 1988 Greater Yellowstone Area fires, Ryan and Amman [7] found that infestations of Douglas-fir beetle (*Dendroctonus pseudotsugae* Hopkins), spruce beetle (*Dendroctonus rufipennis* Kirby), and pine engraver *(Ips pini* Say) increased over time, and suggested that populations might initially increase in fire-injured trees and subsequently spread to uninjured trees. Bark beetles may exploit a temporary window where fire injury reduces tree defenses [4, 8], although there is no evidence for initiation of a large-scale bark beetle outbreak following fire [9].

In fire-injured red pine (*Pinus resinosa* Aiton), resin flow initially decreased by 30%, and attacks by *Ips* spp. were more than twice that of uninjured trees, but after 55 days resin in surviving trees increased [10]. Resin flow of fire-injured ponderosa pine (*Pinus ponderosa* Doug. ex Law) likewise initially decreased with fire injury, and then increased and remained elevated for at least four years [11]. The degree of crown injury in ponderosa pine was associated with more attacks, mostly by secondary bark beetles [5, 12, 13]. Inducible biochemical defenses of lodgepole pine (*Pinus contorta* var. *latifolia* Doug.) are likewise impaired by wildfire injury [14].

The relationships between fire injury and susceptibility to mountain pine beetle (*Dendroctonus ponderosae* Hopkins) attack appear complex. Many early studies observed little post-fire mountain pine beetle activity, suggesting that this insect was not preferentially attracted to fire-injured trees [2, 3, 5, 13, 15]. However, Geiszler et al. [16] found that mountain pine beetle attacked a high proportion of uninjured and lightly injured lodgepole pines in stands that experienced fire. Elkin and Reid [17] observed that mountain pine beetle attacked, but did not prefer, fire-injured trees, and that beetle attacks were more successful in fire-injured than uninjured lodgepole pines at low but not high attack densities. More recent studies have also found that mountain pine beetle attacked fire-injured trees following prescribed fire and thinning treatments [18–20], as well as wildfire [8, 9, 21]. Following prescribed fire in ponderosa pine in northern California, approximately 83% of all tree mortality caused by mountain pine beetle occurred on burned plots [17]. The pattern in which mountain pine beetles respond also depends on their stand-level density [9]. Overall, the post-fire conditions under which mountain pine beetle attacks fire-injured trees, its degree of reproductive success under these variable conditions, and whether wildfire can foster outbreaks are not well understood.

Lodgepole and ponderosa pines are major host species of mountain pine beetles, but they differ substantially in morphology, defensive chemistry, and forest structure. In northern Utah, lodgepole pine occurs at elevations between 2280 and 3140 m, in seral single-age forests generated following stand replacing disturbance, or dual-age forests released by partial survival following disturbance [22–23]. Due to its thin bark, moderate and severe fire injuries are often lethal to lodgepole pine, but survival is usually high following low-severity surface fires [22]. Ponderosa pine in this region (subsp. *scopulorum* [Engelm.]) often occurs in uneven-age forests with heterogeneous structure, and on lower slopes at moderate elevations (~ 2130 to 2745 m) [24, 25]. The thick bark on ponderosa pine allows higher survival following moderate and severe fires [8, 26].

We monitored mountain pine beetle attacks and brood emergence in lodgepole and ponderosa pines for 3 years following a 2007 wildfire in northeastern Utah. Specifically, we examined (1) how the degree and type of fire injury influenced beetle attacks, brood emergence, and subsequent tree mortality, (2) how the number of attacked trees and beetle population level changed over time, and (3) how tree species influenced these relationships.

## Materials and Methods

Permission for field work was obtained from the Roosevelt Ranger District Office, USDA—Forest Service, Ashley National Forest.

### Study Area

**Fig 1:**
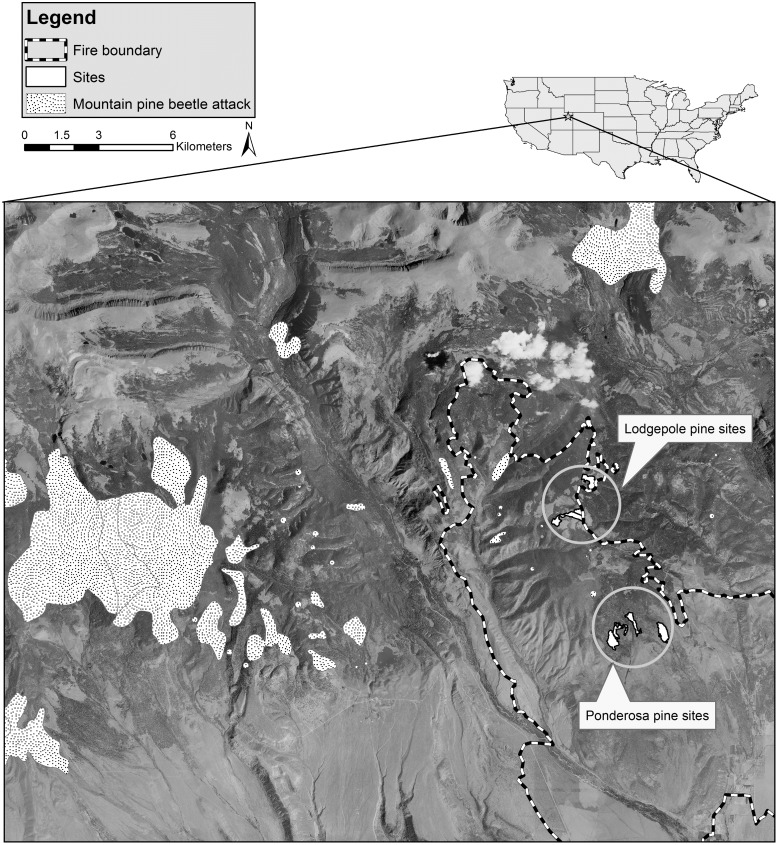
Aerial photograph showing locations of lodgepole and ponderosa pine sites in relation to the Neola North wildfire boundary, and nearest reported mountain pine beetle activity. All sites were affected by the 2007 Neola North fire in northeastern Utah.

The study sites were located within the boundaries of the Neola North Fire, approximately 40 km north of Roosevelt, Utah on the southern slope of the Uinta Mountains (Fig 1). The Aerial image is from the 2006 NAIP (National Agriculture Imagery Program) ortho photo [27], and polygons of mountain pine beetle attack and associated tree mortality in 2006, the year prior to the fire, are from aerial detection surveys [28]. This fire burned approximately 17,700 ha from 29 June to 23 October 2007.

The Neola North Fire was chosen for this study because of the proximity of fire-injured trees to recent mountain pine beetle activity, combined with the absence of an existing outbreak in the immediate affected area (Fig 1). Individual sites within the study area were selected based on the presence of lodgepole or ponderosa pines occurring in a mosaic exhibiting various levels of fire injury, and ideally including uninjured trees. Uninjured trees, where available, provided additional data on beetle response to a continuum of fire injury. Uninjured trees also provided an opportunity to assess if beetle attacks switched from fire injured to uninjured host trees during the timeframe of our study.

Lodgepole pine sites were located on a plateau at elevations between 3,036 and 3,048 m with slopes <10%. Mean annual temperature during the study was 0.6°C (measured on site with HOBO^®^ data logger, Onset Computer Corp, MA) and mean annual precipitation is approximately 75 cm [29]. Soils are deeper and less rocky than in the lower elevation ponderosa pine sites, and have more accumulated organic matter. Surrounding vegetation included 5–10 year-old lodgepole pine regeneration in clearcut sites and nonhost Engelmann spruce-subalpine fir (*Picea engelmannii* Parry ex Engelm. and *Abies lasiocarpa* (Hook.) Nutt.) forests. The burn in lodgepole pine was characterized by active crown fire, with many trees suffering fatal fire injuries. Study sites were located near the burn edge where surface fire provided a mosaic of trees with various levels of injury and a large number of uninjured trees.

Ponderosa pine sites were located on dry ridges and slopes (from 7–45%), with thin, rocky soils, between 2,426 and 2,719 m in isolated stands. Mean annual temperature during the study was 4°C (measured on site with HOBO^®^ data logger, Onset Computer Corp, MA) and mean annual precipitation is approximately 62cm [29]. Surrounding vegetation is characterized by grassland-sagebrush on lower slopes, Douglas-fir (*Pseudotsuga menziesii* (Mirb.) Franco) on sheltered slopes, and lodgepole pine at higher elevations. Virtually all ponderosa pines in our study sites were burned to some degree due to their central location within the burn area. The fire was characterized as a surface fire with occasional passive crowning.

Aerial Detection Surveys from 2006, the year prior to the fire, indicate that epidemic populations of mountain pine beetle were within ~10–15 km of the study area [28] (Fig 1). Pockets of trees with mountain pine beetle attack were within 2 km of the study area. Regionally, the area affected by mountain pine beetle decreased by 37% from 2007 to 2010, but locally, within 5 km of the study area, there was ~20% increase [28].

### Site and Plot Establishment

Four sites each were established in lodgepole (located near 40.6137 N, -110.0410 E) and ponderosa (located near 40.5723 N, -110.0124 E) pine forests within the burn area. Sixteen plot centers were distributed systematically within each site. Lodgepole pine plots were 0.02-ha circular plots. In 39 plots, fewer than five trees with ≥13 cm at DBH were in each plot, so these were expanded to 0.04 ha, and one plot was expanded to 0.08 ha. Ponderosa pine forests were less dense, so larger 0.04-ha circular plots were used. In 27 plots, fewer than five ponderosa pines with diameter at breast height (DBH) ≥13 cm were present, so these plots were expanded to 0.08 ha. In one plot that expansion still did not suffice, so the plot center was moved 20.1 m at a random azimuth.

Lodgepole pine sites had similar tree diameters and heights but varied in tree density, basal area and stand density index (SDI) (S1 Table). Likewise, ponderosa pine sites had minor variation in tree diameter and height, and higher variation in tree density, basal area and SDI. On average, ponderosa pines were larger in DBH than lodgepole pines, had similar heights, and were in less dense stands (S1 Table).

### Fire Injury Measurements

All trees ≥ 13 cm DBH, the typical lower size limit of trees preferred by mountain pine beetle [30], were evaluated for fire injury, height and DBH. Fire injury measurements were collected between June and July 2008, the summer following the fire, for trees considered live prior to the fire. Trees with no fine branches and little to no bark were considered dead prior to the fire and not monitored. Fire injury measurements were collected following methods described by Fowler and Sieg [31] and Hood et al. [32], including indices of both crown and stem injury (Table 1). Tree status (alive or dead) was evaluated based on the presence or absence of living (green) needles in late August or early September of 2008, 2009, and 2010.

**Table 1:** Fire injury measures recorded on each tree.

Variable	Abbreviation	Description
Crown volume scorched	CVS	Percent of crown volume killed by convective heat (red-orange needles)^a^
Crown volume consumed	CVC	Percent of crown volume killed by combustion (black needles)^a^
Total crown damage	TCD	CVC + CVS^a^
Cambium kill rating	CKR	Number of quadrants of the lower bole with dead cambium (0–4)^b^
Bole scorch percentage	BSP	(Mean char height of bole quadrants / total tree height)*100
Bole char index	BCI	Visual estimate of char intensity on four quadrants (0–3)^c^

Note: See Fowler and Sieg [31] and Hood et al. [32] for additional details

^a^ Measured in 10% increments between 10 and 100%, and 1% and 5% below 10%

^b^ Cambium status (live or dead) was evaluated by sampling the phloem tissue by exposing approximately 2.5cm of phloem tissue with a hatchet. Lightly colored, moist, spongy phloem was considered live. Discolored, darkened, resinous, or dry phloem was considered dead. A sample containing both live and dead tissue was considered live.

^c^ Char intensity measured as no visible damage (0), signs of light contact with fire, but bark not completely black and its characteristics are easily discernable (1), bark moderately damaged by fire with a uniformly black color except within fissures, but bark shape and texture remain intact (2), or bark deeply burned and all bark is black with no discernable characteristics (3)

### Monitoring Bark Beetle Population Levels

Four flight intercept traps were deployed in each site to monitor beetle population levels. These unbaited traps consisted of two clear Plexiglas panes (81 H x 41W cm) arranged in a cross pattern with an attached funnel and collection cylinder, similar to those described by Bentz [33]. A small (1.5 cm x 1.5 cm) piece of Hot Shot^®^ No-Pest^®^ strip, containing 2,2-dichlorovinyl dimethyl phosphate, was added to collection cylinders to ensure insects did not escape and to reduce predation. The trap locations were distributed roughly equidistant within a site. Traps were hung between two trees with nylon rope at mid-bole (~6 m), as recommended by Schmitz [34], and sampled weekly.

### Bark Beetle Attack Measurements

Incidence of attack by bark beetles was evaluated for every tree in each plot. In 2008, attack was assessed in June, prior to beetle emergence in July, to measure attacks following the fire in 2007. Subsequent evaluations were conducted in late August or early September of 2008, 2009 and 2010 to tally the current year’s attacks. The fire ignited on 29 June 2007, likely prior to the flight of mountain pine beetle, which is supported by our observation that pitch and frass occurred over charred bark. Each tree bole was examined up to ~6–8 m height for evidence of entrance holes and pitch tubes, the base was examined for frass and boring dust, and sections of bark were removed to validate attacks by various beetle groups. Attacks by bark beetles and woodboring insects were identified by the color, shape, and texture of frass, the color and location of pitch tubes, and the larval and parent galleries under the bark [35, 36]. Current attacks were differentiated from prior-year attacks by the aforementioned criteria and increases in the percent of the bole circumference containing attacks. The extent of mountain pine beetle attack on an individual tree was visually estimated based on the percentage of circumference occupied, in 10% increments. Trees were considered mass attacked when the bole circumference attacked was ≥ 20% and resulted in tree death. Strip attacks had ≤ 20% bole circumference attacked and did not directly result in mortality, and failed attacks had ≤10% bole circumference attacked. Evidence of other bark beetle and woodboring insect species on the bole was recorded as present/absent.

### Mountain Pine Beetle Brood Production

Emergence cages were placed on mass-attacked trees that had experienced varying degrees of fire injury, or were uninjured, to evaluate brood production. Cages constructed of nylon mesh covered a 30.5 cm x 61 cm area, centered at 137 cm height on the north and south aspects of a tree. The outer bark of the caged area was smoothed using a draw shaver to ensure the cages were flush with the bark, and nylon mesh was attached using a staple gun. The bottom portion of the nylon mesh converges in a conical shape, to funnel insects into a centrifuge tube containing Hot Shot^®^ No-Pest^®^ strip (approximately 1 cm x 1 cm containing 2,2-dichlorovinyl dimethyl phosphate). Cages were attached in June of 2008, 2009, and 2010 to different trees each year, prior to mountain pine beetle emergence. Emergence cages were sampled weekly. Each caged tree was evaluated for all fire injury parameters described above to relate brood production to the degree of fire injury. There were 20 caged trees per tree species in 2008, 30 in 2009, and 30 in 2010.

Because very few ponderosa pines within established sites were unburned, and no unburned trees were attacked, we caged 12 attacked trees that were approximately 800 m beyond the burn edge in 2009 and 2010. The off-plot caged unburned trees were within the elevation range of the established ponderosa sites. Following completion of adult emergence, cages were removed and bark underneath the caged area was peeled away to expose galleries. The number of ovipositional galleries in each tree was counted to calculate the ratio of brood to ovipositional galleries.

### Analysis

All analyses were performed in R statistical software v. 2.12.1 and v. 3.2.4 [37]. Normality of all continuous data was evaluated using Q-Q plots, and all models presented in this manuscript reasonably met their underlying assumptions. One-way ANOVA was used to determine if fire injury measures differed between tree species or mountain pine beetle attack type. Correlations between fire injury measures were calculated using both the correlation coefficient R, and the Spearman rank coefficient *R*_*s*_. Linear mixed models using the lmer function in lme4 package (Bates and Maechler 2010) with random error from plot and site, and post-hoc Tukey honestly significant difference (HSD) tests, were performed to determine if the mean level of fire injury on surviving trees differed between years.

Generalized linear mixed models with a Poisson distribution and random error from plot and site were conducted using the glmer function in lme4 package [38]. This test evaluated if numbers of mountain pine beetle and other beetle species caught in flight intercept traps differed between year and tree species, based on a standardized 7-week period during each summer in 2008, 2009 and 2010. The same models were used to determine if mountain pine beetle reproductive measures differed by fire injury, year, or tree species.

Generalized linear mixed models with a binomial distribution and random error from plot and site were used to determine how year affected attack by bark beetles and tree mortality. Likelihood ratio Chi-square tests (LRT) were used to determine the significance of year or fire injury level in these models.

Generalized linear mixed models with a binomial distribution were used to describe how fire-related tree measurements predicted attack by bark beetles and tree mortality. Continuous fire-related tree measurements, TCD, CVS, CVC, and BSP, were modeled with an allowance for a polynomial component, and CKR and BCR were modeled as categorical predictors.

Linear models were used to relate site-level attack to the number of beetles caught in flight traps, site-level fire injury characteristics, and brood emergence within a site. Variance inflation factor diagnostics were generated for covariates within all models to identify the degree to which collinearity increases the standard error. Multicollinearity is generally considered problematic when the variance inflation factor is > 10 [39].

Nonparametric conditional inference trees using binary recursive partitioning were also performed to further determine relevant fire injury parameters associated with mountain pine beetle attack, using the ctree function in the party package [40]. Results are reported in [41].

## Results

### Fire Injury Characteristics

**Fig 2:**
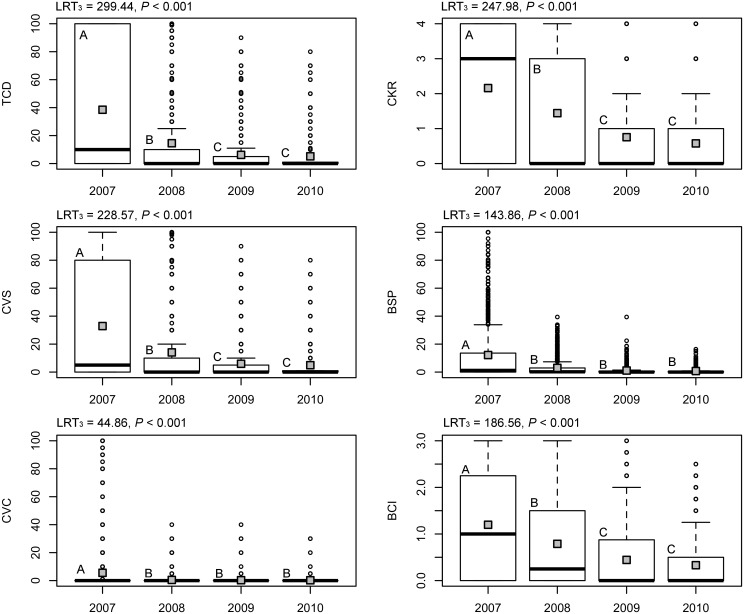
Changes in fire injury distributions among living lodgepole pines over time since fire. Dark lines within boxplots indicate the median and grey boxes indicate mean for six fire injury measurements. Different letters denote significant differences (*α* ≤ 0.05) between years from generalized linear mixed models with Tukey HSD pairwise comparisons. All trees were considered live in 2007.

**Fig 3:**
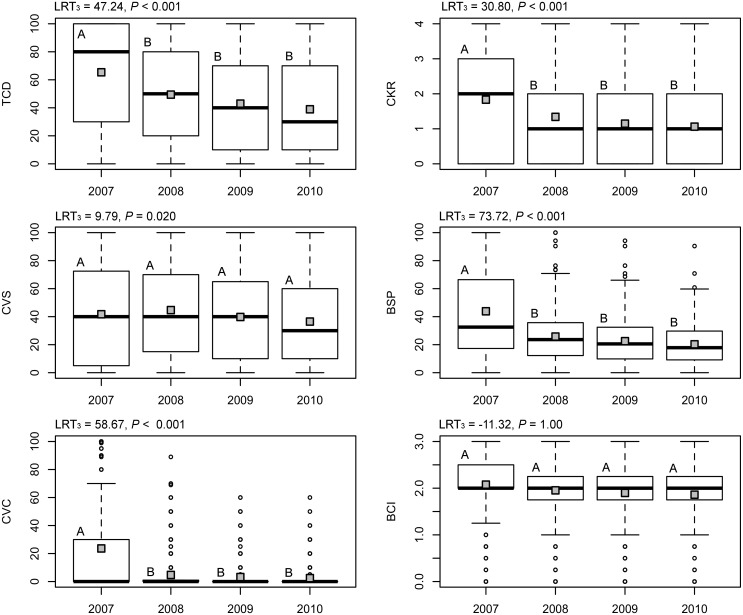
Changes in fire injury distributions among living ponderosa pine over time since fire. Dark lines within boxplots indicate the median and grey boxes indicate mean for six fire injury measurements. Different letters denote significant differences (*α* ≤ 0.05) between years from generalized linear mixed models with Tukey HSD pairwise comparisons. All trees were considered live in 2007.

A total of 870 out of 1173 lodgepole pines, and 572 out of 599 ponderosa pines, had some degree of fire injury. Trees in ponderosa pine sites sustained higher fire injuries to their crown (TCD, CVS, CVC) and outer bark (BSP, BSI) than those in lodgepole pine sites, but less to their cambial tissues, i.e., CKR (Table 2). Over the course of the study, the more severely injured trees died, so the remaining live trees had lower mean fire injury for all fire measures. Thus, a greater proportion of mountain pine beetle attacks tended to occur in trees with lesser fire injury over time. In lodgepole pine, this trend was strongest from 2007 to 2008 (Fig 2). In ponderosa pine, mean fire injury of the remaining live trees declined from 2007 to 2008 for all measures except CVS and BCI, and did not change thereafter (Fig 3). The various fire injury measures were highly correlated within trees, in both species (S2 Table). However, variance inflation factors for covariates within all models were < 3, indicating that muliticollinearity issues were not problematic.

**Table 2:** Comparison of fire injury levels between lodgepole and ponderosa pines using one-way ANOVA.

	All Trees				Attacked Trees (Mass + Strip)				Trees Killed By Fire Injury^1^			
Fire Injury Measure	Lodgepole $\bar{x}$ ± SE	Ponderosa $\bar{x}$ ± SE	*F*_1, 1770_	*P*	Lodgepole $\bar{x}$ ± SE	Ponderosa $\bar{x}$ ± SE	*F*_1, 637_	*P*	Lodgepole $\bar{x}$ ± SE	Ponderosa $\bar{x}$ ± SE	*F*_1, 484_	*P*
TCD	38.5 ± 1.3	65.4 ± 1.5	159.1	<0.001	27.4 ± 2.0	81.2 ± 1.6	369.5	<0.001	76.3 ± 2.0	97.9 ± 1.2	26.3	<0.001
CVS	32.9 ± 1.2	41.7 ± 1.5	19.6	<0.001	25.8 ± 1.9	58.2 ± 2.1	123.5	<0.001	62.5 ± 2.1	20.3 ± 3.7	77.8	<0.001
CVC	5.6 ± 0.6	23.6 ± 1.5	179.5	<0.001	1.6 ± 0.4	23.0 ± 1.5	143.4	<0.001	13.7 ± 1.5	77.6 ± 4.0	306.0	<0.001
CKR	2.2 ± 0.1	1.9 ± 0.1	14.7	<0.001	1.9 ± 0.1	2.1 ± 0.1	1.8	0.181	3.7 ± 0.1	3.0 ± 0.1	32.9	<0.001
BSP	12.2 ± 0.7	43.9 ± 1.4	528.5	<0.001	5.5 ± 0.6	50.6 ± 1.9	779.3	<0.001	27.8 ± 1.6	87.9 ± 2.7	276.8	<0.001
BCI	1.2 ± 0.03	2.1 ± 0.03	314.0	<0.001	1.0 ± 0.1	2.2 ± 0.03	392.9	<0.001	2.1 ± 0.04	2.4 ± 0.04	7.5	<0.001

^1^ Dead trees with fire injury but no mountain pine beetle

Note: Fire injury was measured in 2008, the year after the 2007 fire. All trees includes live trees, attacked trees, and trees that died during the study. Attacked trees include only trees with mass or strip attacks from mountain pine beetle, and excludes trees in which attacks failed. See Table 1 for descriptions of fire injury measures

### Bark Beetle Population Levels

**Fig 4:**
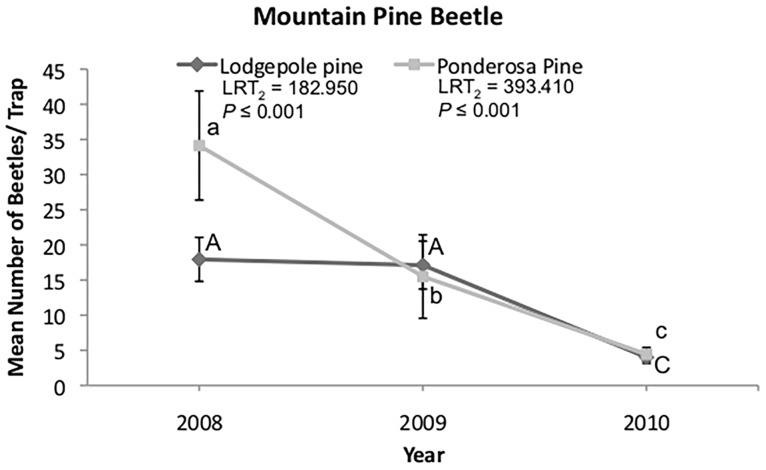
Mean ± SE numbers of mountain pine beetles caught in unbaited flight intercept traps. Data reflect 3 years post-fire in lodgepole and ponderosa pine sites. Different letters denote significant differences (*α* ≤ 0.05) between years within a species from generalized linear mixed models with Tukey HSD pairwise comparisons.

Numbers of mountain pine beetles caught in flight intercept traps declined between 2008 and 2010 in both lodgepole (*z* = 10.846, *P* ≤ 0.001) and ponderosa (*z* = 15.820, *P* ≤ 0.001) pine sites (Fig 4; S3 Table). The total number of mountain pine beetles caught did not differ between tree species (LRT_1_ = 0.270, *P* = 0.601), but there was a significant interaction between tree species and year (LRT_1_ = 40.424; *P* ≤ 0.001). The number of mountain pine beetles caught in lodgepole pine sites was similar the first two years following wildfire, and then declined in 2010 (Fig 4). In ponderosa pine, the number of mountain pine beetles declined each year.

The number of *Ips* spp. caught in traps declined between 2008 and 2009 in both tree species (S1 Fig; S3 Table). There were more *Ips* spp. caught in lodgepole than ponderosa pine sites (LRT_1_ = 6.494, *P* = 0.011). Other Scolytinae species caught in flight traps, from most to least common, were twig beetles (*Pityogenes* spp., *Pityophthorus* spp., *Pityokteines* spp.), red turpentine beetle (*Dendroctonus valens* LeConte), lodgepole pine beetle (*Dendroctonus murrayanae*, Hopkins), and *Hylastes* spp. Hereafter, the term ‘other Scolytinae’ refers to those other than *D*. *ponderosae* or *Ips*. spp. Over 3 years, more of these other Scolytinae were caught in lodgepole than ponderosa pine sites (LRT_1_ = 8.573, *P* = 0.003), and there was a significant tree species by year interaction (LRT_2_ = 300.690, *P* ≤ 0.001). Catches of other Scolytinae differed between each year, in both tree species (S1 Fig).

### Mountain Pine Beetle Attack Patterns

#### Lodgepole pine

**Fig 5:**
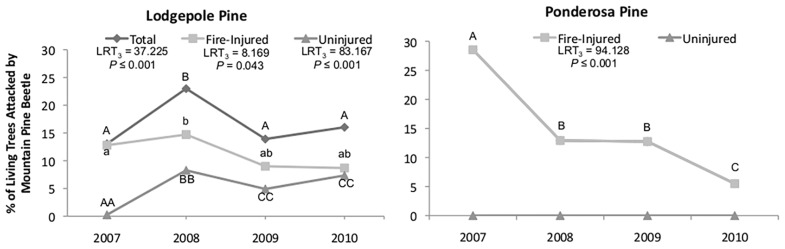
Percent of lodgepole (left) and ponderosa (right) pines that were mass- or strip- attacked by mountain pine beetles over four years. The fire occurred in 2007, prior to beetle flight. Attacks occurred on uninjured and fire-injured lodgepole pine, whereas only fire-injured ponderosa pines were attacked. Different letters denote significant differences (*α* ≤ 0.05) between years within a tree category from generalized linear mixed models with Tukey HSD pairwise comparisons.

Mountain pine beetle attacked 46% of the 1173 lodgepole pines evaluated between 2007 and 2010, with 84% of these attacks occurring by the end of 2008, the year after the fire (Table 3). During 2008, 22% of living lodgepole pines were mass-or strip-attacked, a higher percentage than in any other year (Fig 5). This increase was largely attributable to an increase in attacks on uninjured trees (Fig 5). During the year of the fire, 2007, when almost all attacks were on injured trees, 33% of attacks resulted in mass attack (Table 3). During the ensuing years, when beetles attacked both fire-injured and uninjured lodgepole pines, only 15% of attacks resulted in mass attack, and the percentage of failed attacks rose from 22% to 33%. No uninjured trees were mass attacked in 2007 or 2008. Among uninjured trees in 2009 and 2010, however, 28% and 26%, respectively, were successfully mass attacked.

**Table 3:** Number of lodgepole and ponderosa pines attacked by mountain pine beetle (Top) and *Ips* spp., other Scolytinae and woodborers (Bottom) each year and all years pooled.

Year	Lodgepole Pine (*n* = 1173)				Ponderosa Pine (*n* = 599)			
	**Mountain Pine Beetle Attack**				**Mountain Pine Beetle Attack**			
	All Attacks	Mass Attacks	Strip Attacks	Failed Attacks	All Attacks	Mass Attacks	Strip Attacks	Failed Attacks
2007	197	65	88	44	241	83	88	70
2008	280	22	167	167	82	18	35	29
2009	129	37	48	48	68	26	18	24
2010	108	20	52	52	24	8	8	8
Pooled	546	144	250	250	337	135	110	92
	**Attack From Additional Beetle Species**				**Attack From Additional Beetle Species**			
	*Ips* spp	Other Scolytinae	Woodborers		*Ips* spp	Other Scolytinae	Woodborers	
2007	287	156	-		37	17	-	
2008	112	74	-		1	1	-	
2009	68	32	-		1	6	-	
2010	38	4	-		0	23	-	
Pooled	461	259	446		39	45	335	

Note: Some trees were attacked in more than one year, thus pooled attacks are not necessarily equal to the sum of the attacks each year. The value of *n* refers to the total number of attacked and unattacked trees. Woodborers were not tabulated annually, thus only pooled values are displayed.

**Fig 6:**
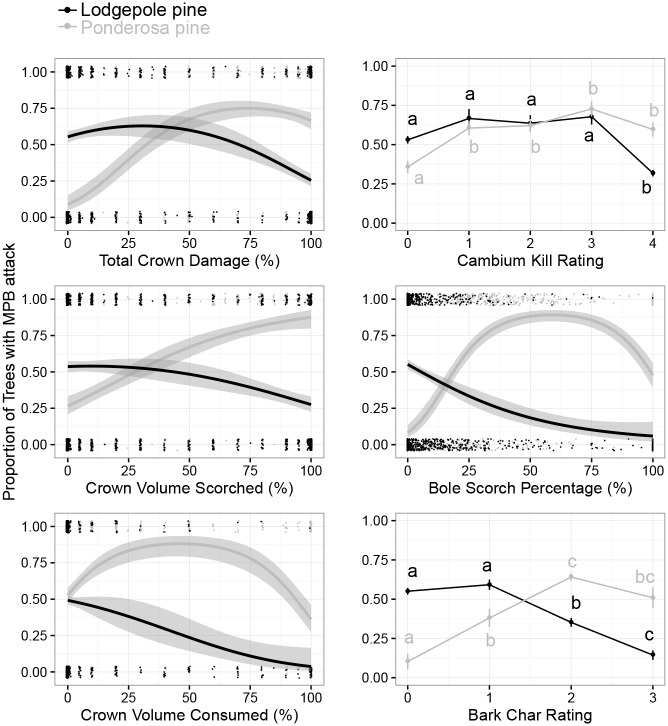
Relationship between proportion of trees with mountain pine beetle attacks (all attack types) and fire injury. Generalized linear models representing the probability of MPB attack on lodgepole and ponderosa pines within 3 years post-fire for four continuous and two categorical measures of fire injury. Models for each continuous measure were allowed a polynomial term, although in some cases these terms were not significant. For categorical measures, different letters indicate significant differences (*α* ≤ 0.05) among severities of fire injury from Tukey HSD pairwise comparisons. All data were modeled with a binomial distribution and all models were globally significant as measured with likelihood ratio tests. Model parameters in S4 and S5 Tables.

Significant relationships between degree of fire injury and incidence of mountain pine beetle attack were observed for both continuous and categorical burn metrics (S4 and S5 Tables). The incidence of attack showed a curvilinear relationship with TCD (the combination of CVS and CVC), a declining relationship with BSP, and an initially level but subsequently declining relationship with CKR and BCI (Fig 6).

Lodgepole pines that underwent mass attacks had greater fire injury than those that experienced strip attacks, or on which attacks failed, for all measures, and this difference was significant for all but CVC (Table 4). For example, trees that were mass attacked had TCD and CVS over twice that of trees on which attacks failed, and BSP over 30% higher than that of trees on which attacks failed.

**Table 4:** One-way ANOVA results comparing mean fire injury among mountain pine beetle attack types on lodgepole (left) and ponderosa (middle) pines, and between tree species (right).

	Lodgepole Pine					Ponderosa Pine					Species Comparison					
	Mass	Strip	Failed	*F*_*2*,*540*_	*P*	Mass	Strip	Failed	*F*_2, 331_	*P*	Mass		Strip		Failed	
	$\bar{x}$ ± SE	$\bar{x}$ ± SE	$\bar{x}$ ± SE			$\bar{x}$ ± SE	$\bar{x}$ ± SE	$\bar{x}$ ± SE			*F*_1,277_	*P*-value	*F*_1,502_	*P*-value	*F*_1,277_	*P*-value
TCD	48.4 ± 3.9 a	15.3 ± 1.8 b	20.3 ± 2.9 b	39.0	≤ 0.001	88.0 ± 2.0 a	72.8 ± 2.4 b	68.4 ± 3.4 b	21.7	≤ 0.001	80	≤ 0.001	584.9	≤ 0.001	240.9	≤ 0.001
CVS	45.4 ± 3.7 a	14.4 ± 1.7 b	17.8 ± 2.6 b	39.6	≤ 0.001	53.0 ± 3.2 a	64.5 ± 2.6 b	51.7 ± 3.7 a	4.8	0.009	2.4	0.124	438.3	≤ 0.001	156.4	≤ 0.001
CVC	3.0 ± 0.9 a*	0.8 ± 0.3 a*	2.6 ± 1.0 a	3.3	0.039	35.0 ± 3.4 a	8.3 ± 1.6 b	16.7 ± 3.3 b	25.2	≤ 0.001	86.4	≤ 0.001	78.8	≤ 0.001	29.8	≤ 0.001
CKR	2.4 ± 0.2 a	1.7 ± 0.1 b	1.4 ± 0.1 b	13.8	≤ 0.001	2.4 ± 0.1 a	1.8 ± 0.1 b	1.9 ± 0.1 b	6.8	≤ 0.001	0.1	0.721	1.8	0.182	11.7	≤ 0.001
BSP	9.1 ± 1.3 a	3.4 ± 0.4 b	6.3 ± 1.2 a	10.4	≤ 0.001	60.8 ± 2.8 a	38.1 ± 1.6 b	40.4 ± 3.2 b	26.3	≤ 0.001	295.2	≤ 0.001	1245.0	≤ 0.001	256.7	≤ 0.001
BCI	1.1 ± 0.1 a	0.9 ± 0.1 b	0.8 ± 0.1 b	4.0	0.020	2.3 ± <0.1 a	2.2 ± <0.1 ab	2.1 ± <0.1 b	6.0	0.003	160.6	≤ 0.001	332.4	≤ 0.001	259.4	≤ 0.001

Note: Different letters indicate significant difference (*α* ≤ 0.05) in mean fire injury between attack types based on Tukey HSD contrasts within tree species.

* Denotes contrast that was marginally significant (*P* = 0.055).

The likelihood of mass attack was positively related to CVS, CKR, DBH, and negatively to BCI in mixed models (S6 Table). Mass attacks varied in a complex manner with year due to the negative interaction with CKR. The likelihood of strip attacks was positively related to CKR and DBH, and negatively with TCD, BSP and the interaction of CKR and Year (S6 Table). While not significant as a part of the full models, an interaction of fire injury with time resulted in strip and mass attacks occurring on lodgepole pines with less TCD (LRT _3_ = 49.344, *P* < 0.001), CVS (LRT_3_ = 56.752, *P* < 0.001), BSP (LRT_3_ = 26.502, *P* < 0.001), and BCI (LRT_3_ = 34.738, *P* < 0.001) from 2008 to 2010.

#### Ponderosa pine

Mountain pine beetle attacked 56% of 599 ponderosa pines between 2007 and 2010, with 72% of these occurring in 2007, the year of the fire (Fig 5, Table 3). During 2007, 34% of the attacks on ponderosa pine resulted in mass attacks, 37% resulted in strip attacks, and 29% resulted in failed attacks. The proportion of ponderosa pines that were attacked declined from 2007 to 2008, and from 2009 to 2010 (Fig 5). After four years, 23% of all ponderosa pines had been mass attacked, 18% had been strip attacked, and 15% experienced failed attacks (Table 3). None of the 27 uninjured ponderosa pines were attacked.

Ponderosa pines that were attacked by mountain pine beetle had significantly greater fire injury to their crowns and outer bark than attacked lodgepole pines, but equivalent levels of cambial injury as lodgepole pines (Table 2). For example, ponderosa pines that underwent mass or strip attacks had experienced over twice the degree of TCD, CVS, and BCI, 9.2 times the degree of BSP, and 14.3 times the degree of CVC as lodgepole pines that underwent mass or strip attacks.

Significant relationships between degree of fire injury and incidence of mountain pine beetle attack were observed for both continuous and burn categorical metrics (S4 and S5 Tables). The incidence of attack showed a curvilinear relationship with TCD, BSP, and BCR, and an initially increasing but subsequently leveling relationship with CKR (Fig 6).

Similar to lodgepole pine, ponderosa pines that underwent mass attacks had greater fire-injury than those with strip or failed attacks for most measures (Table 4). For example, ponderosa pines that were mass attacked had CVC over twice as high, BSP 34% higher, and TCD 22% higher than those on which attacks failed.

The likelihood of mass attack was positively related to TCD, CKR, and DBH in mixed models, and varied significantly by year (S7 Table). Relative to the number of remaining living ponderosa pines, 2009 had the highest frequency of mass attacks, although 2007 had the highest number of mass attacks (Table 3). The likelihood of strip attacks was positively related to CVS and DBH, and decreased each year in mixed models (S7 Table). While not significant in the full models, mountain pine beetle attacks (strip + mass) were influenced by an interaction of fire injury with time that resulted in attacks occurring on ponderosa pines with lower TCD (LRT_3_ = 14.722, *P* = 0.002), and fluctuated over time with BCI (LRT_3_ = 14.103, *P* = 0.003), and CKR (LRT_4_ = 18.684, *P* < 0.001).

### Site-Level Mountain Pine Beetle Attack Patterns Over Four Years

Over the course of four years, the number of trees attacked by mountain pine beetles within a site was positively related to the total number caught in flight intercept traps in lodgepole (*R*^2^ = 0.418, F_1,10_ = 7.179, *P* = 0.023) and ponderosa (*R*^2^ = 0.754, F_1,10_ = 30.59, *P* ≤ 0.001) pines, although trends were not evident within individual years. The number of living lodgepole pines that had some degree of fire injury at the beginning of each year influenced the number of trees that mountain pine beetle attacked (*R*^2^ = 0.497, F_1,10_ = 13.81, *P* = 0.002). Attack frequency declined once the proportion of lodgepole pine with fire injury per site exceeded 0.93.

### Mountain Pine Beetle Reproduction

#### Lodgepole pine

The presence of burn within the caged area reduced the number of ovipositional galleries and total adult emergence (S8 Table). The density of ovipositional galleries per caged area increased from 2008 to 2010 (Table 5), while the mean number of emerged adults and emergence to oviposition ratios varied among years, being highest in 2009 and lowest in 2010. However, these relationships did not scale up to the whole-tree fire injury measures. Total adult emergence in lodgepole pine did not differ between fire-injured and uninjured trees (*z* = 1.555, *P* = 0.120). Mean adult emergence within a site was positively correlated with the number of mountain pine beetle mass and strip attacks in the previous year (*R*^*2*^ = 0.569, *F*_1,10_ = 13.19, *P* = 0.005). Increased number of mountain pine beetle ovipositional galleries within the caged area increased total adult emergence (*z* = 21.890, *P* ≤ 0.001), but decreased the ratio of emergence to oviposition (*t* = -2.872, *P* = 0.005). Increasing numbers of emerged *Ips* spp. was associated with increased adult mountain pine beetle emergence. Conversely, increasing numbers of other Scolytinae was associated with decreased adult emergence and number of ovipositional galleries (S8 Table). The co-occurrence of mountain pine beetle and *Ips* spp. tended to be associated with more strip attacks in fire-injured trees, and fewer mass attacks. Mountain pine beetle emergence was greater in lodgepole pine with *Ips* spp., regardless of fire injury (S8 and S9 Tables).

**Table 5:** Comparison of mountain pine beetle reproductive measures from caged trees between years and tree species using Tukey HSD contrasts from mixed models.

	Lodgepole Pine			Ponderosa Pine			Tree Species Comparison					
Year	Emerging Beetles	Ovipositional Galleries	Emerging Beetles / Ovipositional Galleries	Emerging Beetles	Ovipositional Galleries	Emerging Beetles / Ovipositional Galleries	Emerging Beetles		Ovipositional Galleries		Emerging Beetles / Ovipositional Galleries	
	$\bar{x}$ ± SE	$\bar{x}$ ± SE	$\bar{x}$ ± SE	$\bar{x}$ ± SE	$\bar{x}$ ± SE	$\bar{x}$ ± SE	*z*-value	*P*-value	*z*-value	*P*-value	*z*-value	*P*-value
2008	293 ± 44 a	124 ± 9 a	2.8 ± 0.5 ab	281 ± 45 a	64 ± 5 a	2.8 ± 0.5 a	1.424	0.691	11.786	≤ 0.001	0.000	1.000
2009	473 ± 53 b	146 ± 6 b	4.2 ± 1.1 a	95 ± 16 b	114 ± 6 b	0.8 ± 0.1 b	47.534	≤ 0.001	6.677	≤ 0.001	4.703	≤ 0.001
2010	171 ± 32 c	160 ± 9 b	1.0 ± 0.1 b	22 ± 4 c	100 ± 6 b	0.2 ± 0.1 b	31.108	≤0.001	12.811	≤ 0.001	1.010	0.906

Note: different letters within a column denote significant differences (*α* ≤ 0.05) between years. Includes both fire injured and uninjured trees.

#### Ponderosa pine

Overall, fire injury decreased the density of attack but increased the total number of emerged beetles in ponderosa pine (S8 Table). The number of ovipositional galleries constructed by mountain pine beetle was negatively related to BSP, and the presence of burn within the caged area (S8 Table). However, total adult emergence in ponderosa pine increased with the presence of burn within the caged area, and while not significant within the full model, increased with TCD (*z* = 2.917, *P* = 0.004). There were significantly more ovipositional galleries in 2009 and 2010 than 2008, while the number of emerged adults and the ratio of emergence to oviposition declined significantly each year (Table 5, S8 Table). The number of emerged mountain pine beetles over three years in fire-injured ponderosa pine averaged 123.0 ± 17.1 adults per m^2^, and was significantly lower in uninjured ponderosa pine (*z* = 13.550, *P* < 0.001) with only 46.6 ± 11.3 adults per m^2^. Mean adult emergence within a site was positively correlated with the number of mountain pine beetle mass and strip attacks in the previous year (*R*^*2*^ = 0.68, *F*_1,10_ = 20.90, *P* ≤ 0.001). Increased numbers of mountain pine beetle ovipositional galleries increased the total adult emergence (*z* = 1.965, *P* = 0.049), but decreased the ratio of emergence to ovipositonal galleries (*t* = -2.221, *P* = 0.029).

There were fewer ovipositional galleries per caged area in ponderosa pine than in lodgepole pine over all three years (*z* = - 7.270, *P* < 0.001) and within each year (Table 6). Total adult emergence in ponderosa pine was significantly less than in lodgepole pine (*z* = -6.751, *P* < 0.001). However, total adult emergence did not differ between tree species in 2008 (Table 5). Similar to lodgepole pine, increasing numbers of emerging *Ips* spp. beetles was associated with increased mountain pine beetle emergence in ponderosa pine, while increased numbers of other Scolytinae was associated with decreased emergence and number of ovipositional galleries (S5 Table). Mountain pine beetle emergence was significantly higher in fire-injured ponderosa pines with *Ips* spp. present (S9 Table).

**Table 6:** Number of living fire-injured and uninjured lodgepole and ponderosa pines remaining each year.

	Lodgepole pine		Ponderosa pine	
Year	Fire-injured	Uninjured	Fire-injured	Uninjured
2007	870	303	572	27
2008	519	303	383	27
2009	312	299	318	27
2010	207	242	291	27

### Tree Mortality

#### Lodgepole pine

**Fig 7:**
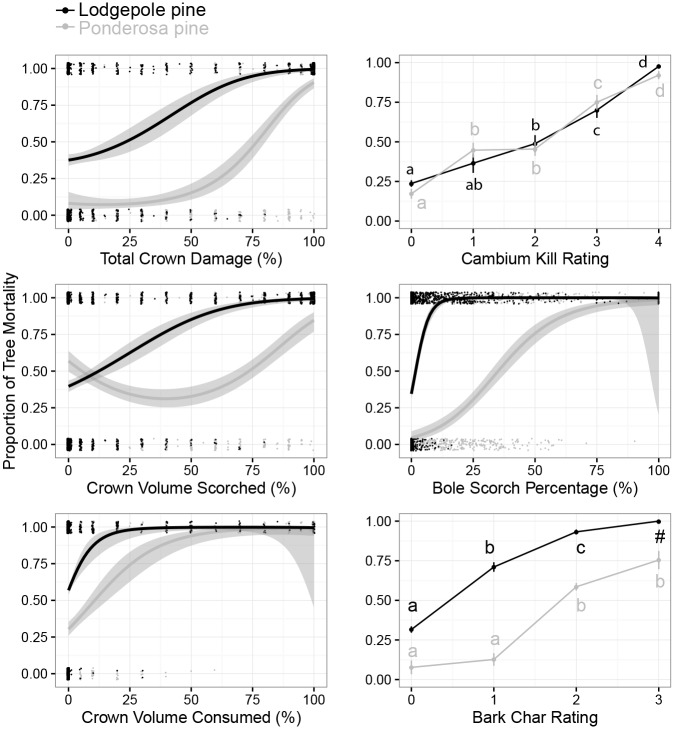
Relationships between fire injury measures and tree mortality. Generalized linear models representing the probability of mortality of lodgepole and ponderosa pines within 3 years post-fire for four continuous and two categorical measures of fire injury. Models for each continuous measure were allowed a polynomial term, although in some cases these terms were not significant. For categorical measures, different letters indicate significant differences (*α* ≤ 0.05) among severities of fire injury categories from Tukey HSD pairwise comparisons. All data were modeled with a binomial distribution and all models were globally significant as measured with likelihood ratio tests. Model parameters in S4 and S5 Tables.

The combination of fire injury and bark beetle attack led to 62% lodgepole pine mortality after four years (Table 6). Combined mortality increased with the degree of fire injury for all measures (Fig 7), and any degree of fire injury increased the probability of mortality (*z* = 12.089, *P* ≤ 0.001). Of the 198 strip-attacked lodgepole pines from 2007 to 2009, 78% were dead by 2010. Of the 159 uninjured trees that were attacked, 37% died by 2010, but only 18% died the year following attack.

#### Ponderosa pine

The combination of fire injury and bark beetle attack led to 51% ponderosa pine mortality after four post-fire years (Table 6). Tree mortality increased with the degree of fire injury for all measures except CVS (Fig 7). Ponderosa pines that died from fire injury alone had greater mean fire injuries than lodgepole pine that died from fire alone, with the exceptions of CKR and CVS (Table 2). Ponderosa pines with mountain pine beetle attack were significantly more likely to die by 2010 than those without attack (*P* ≤ 0.001, *z* = 7.711). Attack during 2007 was positively related to ponderosa pine mortality in the following year (*P* ≤ 0.001, *z* = 5.816). Of 102 strip-attacked ponderosa pines between 2007 and 2009, 60% were dead by 2010.

## Discussion

Mountain pine beetle successfully attacked, reproduced in, and caused mortality to fire-injured ponderosa and lodgepole pines, but there were substantial differences in attack progression and reproductive success between tree species. Despite reproducing in both fire-injured and uninjured trees, beetle populations ultimately declined, as expressed as number of flying beetles, attacked trees, and emerging brood. These results support studies that show both an affinity of mountain pine beetle to fire injured trees and the limitations of this resource for sustained reproduction [8, 9, 42]. They further demonstrate the influence of tree species and fire injury level on mountain pine beetle attack behavior and population response to disturbance.

In our study, both lodgepole and ponderosa pines were affected by the same wildfire. Although the two species were not growing in the same stands, beetle population pressure was similar among sites of both tree species and pre-fire populations appeared to be endemic locally, despite neighboring epidemics. Beetle attack trends, however, differed substantially between tree species. In general, ponderosa pines that were attacked by mountain pine beetle had greater fire injury than lodgepole pines that were attacked (Table 2). The thicker bark of ponderosa pine allows it to sustain greater external fire injury [8] without being physiologically impaired. This can be seen by the overall lower cambial injury (CKR) in ponderosa than lodgepole pine despite the higher burn intensity it experienced by all other crown and outer bark measures. Thus, more external fire injury may be required to reduce both the defenses and nutritional quality of ponderosa pine. As in similar studies, lodgepole pines with severe fire injury were attacked less frequently [9, 21], although elevated beetle populations may increase the breadth of injuries attacked [9]. For a given level of fire injury, a higher proportion of lodgepole than ponderosa pine were killed (Fig 7). Thus, four years post-fire the remaining live ponderosa pines had greater fire injuries than remaining live lodgepole pines (Figs 2 and 3).

In both ponderosa and lodgepole pine, trees that underwent mass attacks had greater fire injury than did those that sustained strip-kill or on which attacks failed (Table 4). This suggests that fire injury makes trees more attractive, susceptible, or both to mountain pine beetle. Tree species may respond differently, and injury to the bole and crown may also differentially affect beetle response. In lodgepole pine, both mass and strip attacks were more likely with low to moderate bole damage (BCI, BSP, CKR), and mass attacks were also more likely with high crown scorch (CVS). In ponderosa pine, both attack types were more likely with high crown damage (TCD), and mass attacks were also more likely with high levels of bole damage (CKR).

Emergence of mountain pine beetle adults was greater in fire-injured lodgepole pine than ponderosa pine (Table 5), and may reflect the higher burn intensity in ponderosa pine. However, the number of emerged adults in 2008, the year after the fire, was similar between tree species, suggesting that recently fire-injured trees of both species make suitable hosts. Others have reported similarly high emergence in lodgepole pine [9] and low emergence in ponderosa pine [8]. The latter was likely due to substantial competition from *Dendroctonus brevicomis* LeConte, which was not present at our study sites. These results support prior observations that post-fire, mountain pine beetle faces a trade-off between host quality, that decreases with fire injury to the bole [16, 9, 43], and host susceptibility, that increases with fire injury [14]. Colonizing beetles adjust their attack densities to the level of a tree’s resistance [9], which caused the higher attack densities, and hence greater intraspecific competition [35], to occur in hosts with less fire injury. Conversely, there were higher numbers of secondary beetles, and hence higher interspecific competition, in more heavily injured hosts, as also seen by others [8, 9]. The degree of fire injury that allows for successful colonization by mountain pine beetle varies with tree species, and in ponderosa pine that degree may not be the most optimal for reproduction.

Spatial and temporal scale also influences beetle emergence. For example, adult brood emergence from lodgepole pine decreased with the degree of burn injury within the caged area sampled (S8 Table), but was not affected by whole-tree measures of fire injury, suggesting that microsite within a tree bole affects brood development. Additionally, the number of attacked trees in a site was higher in years with larger numbers of flying beetles. The pattern of suitable fire-injured hosts within stands influences the number of flying beetles, which in turn affects the number of attacks and subsequent adult emergence. Hence, mountain pine beetle emergence was positively related to the number of attacked trees the prior year. Secondary bark beetles add another level of complexity to post-fire dynamics [42, 44].

Processes occurring at the individual tree scale influenced stand-level dynamics and the progression of attacks over time. In lodgepole pine stands, mountain pine beetles initially attacked and reproduced successfully in fire-injured trees, attacked a greater number of trees the following year, and expanded their attacks to include uninjured trees as the pool of injured trees diminished. None of the uninjured lodgepole pines that beetles entered in 2008 were successfully mass attacked, but successful attacks on uninjured trees occurred during the next two years. In years when mountain pine beetle attacked uninjured trees, however, the overall incidence of attack failure was high. This suggests that exploitation of susceptible fire-injured lodgepole pines generated sufficient population levels to approach but not surpass the eruptive threshold [45].

In ponderosa pine stands, hosts with high TCD and CKR, which were associated with mass attacks in 2007, were plentiful during the year of the fire but became increasingly rare as they died from fire injury and beetle attack. The number of trees that beetles attacked decreased thereafter, causing higher attack densities within fewer hosts, higher intraspecific competition, and lower ratios of emerging adults per ovipositonal gallery (Table 5). Ponderosa pines with low fire injuries remained plentiful, but few were attacked, similar to observations by Davis et al [8]. This suggests that exploitation of susceptible fire-injured ponderosa pines during the first post-fire year did not generate sufficient population levels to succeed in subsequent years. We cannot infer how a mosaic of fire injuries that included more lightly injured and uninjured ponderosa pines would have influenced the course of attacks relative to lodgepole pine. Other factors arising from elevation differences [46], slope [47], past biotic or abiotic disturbances [48,49], forest management and stand structure [50], and varying levels of root damage experienced during the fire [5] may contribute to differential susceptibility between these two tree species.

The patterns of attack progression that we observed suggest that mountain pine beetle requires several conditions to spread from fire-injured trees to uninjured trees: 1) existing population of beetles to attack fire injured trees 2) a sufficient number of suitable fire-injured hosts 3) a sufficient number of uninjured hosts adjacent to injured hosts 4) reproduction in the fire-injured trees that sustains or increases population size 5) weather conditions conducive for beetle overwintering and development. These conditions may be most frequent along burn edges [9] and in areas of mixed-severity burn. Most attacks in this and other studies occurred within 1–2 years following fire [6, 8, 13], suggesting bark beetles have a short window in which to exploit fire-injured trees and shift to uninjured trees, in part because surviving fire-injured trees may be well-defended due to increased resin flow and resin ducts that can persist for multiple post-fire years [11, 51]. Shifting attacks to well-defended trees is density dependent and indicative of incipient-epidemic levels [45]. Despite the initial increase in the number of lodgepole pines killed per year and the expanded range of trees successfully attacked, decreases in mountain pine beetle within-tree per-capita reproductive success, increased rates of attack failure, and lower numbers of flying beetles suggest populations were insufficient to generate transitions to full epidemics. We do not know how long fire injury influences tree defenses, so future research should evaluate the recovery times of different tree species.

In summary, fire-injured trees provide mountain pine beetle with a temporary pool of weakened trees, as found in previous studies [8, 14]. Tree species and fire injury interact to affect attack behavior and reproductive success. Mountain pine beetle initially exploited fire-injured lodgepole and ponderosa pines, and reproductive success in both species was similar the first post-fire year. In lodgepole pine, uninjured trees were then successfully attacked although few mass attacks and more failed attacks occurred in subsequent years, resulting in local population decline. In ponderosa pine, despite good reproductive success the first post-fire year, beetle populations immediately declined. It is unclear what role a lack of uninjured and lightly injured trees played in this decline, other than that the few available uninjured ponderosa pines were not attacked over four years, including in 2008 when mountain pine beetle populations were twice as high as in lodgepole pine sites. The immediate population increase in lodgepole pine suggests it is plausible that fire injury could precipitate epidemic levels, but also that substantial barriers reduce this likelihood. These barriers include interspecific competition, host resistance, substrate quality, availability of susceptible hosts, and additional region-wide favorable conditions arising from weather, forest structure or both [9]. This study and others suggest that mountain pine beetle be considered in post-fire management, but that wildfire is not likely to cause transitions into new outbreaks [8, 9, 21, 52], due to the ‘pulsed resource’ [8] nature of fire-injured trees. Additional research should focus on mountain pine beetle response post-fire, in particular on mechanisms of attraction to and physiology of fire-injured trees, the role of beetle immigration, and potential for landscape-level spread beyond fire boundaries when other drivers such as drought and high temperature are present.

## Supporting Information

S1 Fig Mean numbers of *Ips* spp. and other Scolytinae beetles caught in unbaited flight intercept traps. (TIF)

S1 Table Mensurational characteristics of lodgepole and ponderosa pines in study sites. (DOCX)

S2 Table Correlations between fire injury measures within lodgepole and ponderosa pines. (DOCX)

S3 Table Model parameters for the number of bark beetles caught in flight intercept traps. (DOCX)

S4 Table Model parameters for binomial models of fire injury categories predicting tree mortality and MPB attack. (DOCX)

S5 Table Likelihood ratio tests for binomial models of fire injury categories predicting tree mortality and MPB attack. (DOCX)

S6 Table Parameters providing the best fit for the likelihood of attack by mountain pine beetle on lodgepole pine. (DOCX)

S7 Table Parameters providing the best fit for the likelihood of attack by mountain pine beetle on ponderosa pine. (DOCX)

S8 Table Parameters for mountain pine beetle reproduction models. (DOCX)

S9 Table Comparisons of mountain pine beetle performance in presence or absence of *Ips*. (DOCX)
