# Supplementary figures and images for: Mountain Pine Beetle Dynamics and Reproductive Success in Post-Fire Lodgepole and Ponderosa Pine Forests in Northeastern Utah
Source: PLoS One. 2016 Oct 26;11(10):e0164738. doi: 10.1371/journal.pone.0164738 (PMC5082653; doi:10.1371/journal.pone.0164738)

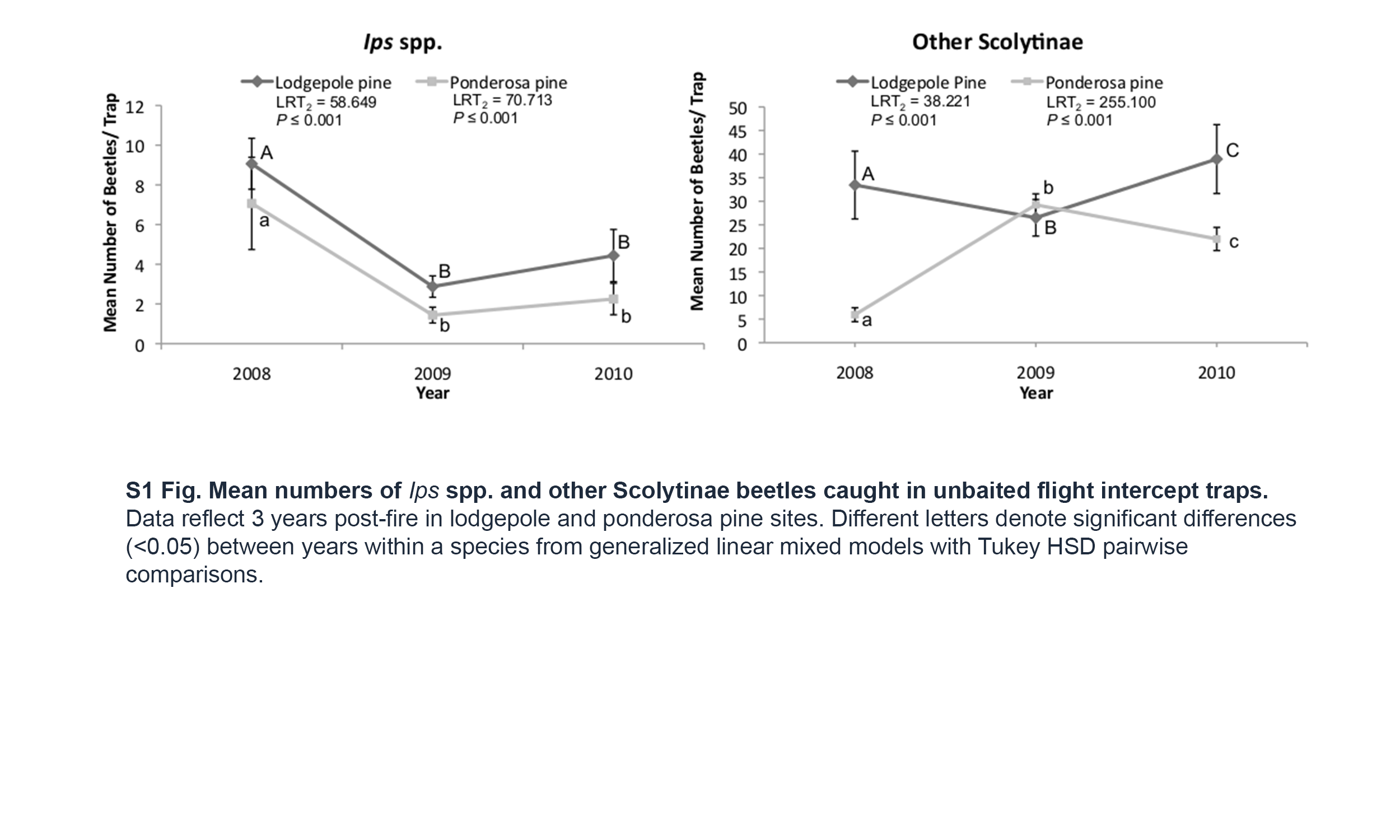

Supplement: S1 Fig — (TIF) [file pone.0164738.s001.tif]
