# Supplementary material for: Mountain Pine Beetle Dynamics and Reproductive Success in Post-Fire Lodgepole and Ponderosa Pine Forests in Northeastern Utah
Source: PLoS One. 2016 Oct 26;11(10):e0164738. doi: 10.1371/journal.pone.0164738 (PMC5082653; doi:10.1371/journal.pone.0164738)
Supplement: S1 Table — (DOCX) [file pone.0164738.s002.docx]

**S1 Table. Mean ± SE of tree mensurational characteristics for sites in lodgepole and ponderosa pine.**

| **Species** | **Site** | **DBH**  **(cm)** | **Height**  **(m)** | **Density (trees/ha)** | **BA**  **(cm^2^)** | **SDI** |
| --- | --- | --- | --- | --- | --- | --- |
| Lodgepole Pine | 1 | 25.1 ± 0.8 | 14.2 ± 0.3 | 437 ± 58 | 635.1 ± 82.4 | 183 ± 24 |
|  | 2 | 25.7 ± 0.7 | 14.9 ± 0.3 | 518 ± 48 | 774.0 ± 51.5 | 222 ± 15 |
|  | 3 | 24.9 ± 0.8 | 15.6 ± 0.2 | 698 ± 37 | 971.7 ± 47.6 | 283 ± 13 |
|  | 4 | 26.9 ± 1.0 | 14.4 ± 0.3 | 675 ± 57 | 1043.1 ± 63.7 | 296 ± 16 |
| Ponderosa Pine | 1 | 35.6 ± 1.9 | 13.5 ± 0.4 | 134 ± 14 | 409.7 ± 38.7 | 101 ± 9 |
|  | 2 | 40.3 ± 1.7 | 13.9 ± 0.4 | 166 ± 19 | 621.1 ± 60.4 | 148 ± 14 |
|  | 3 | 39.4 ± 1.3 | 15.4 ± 0.4 | 153 ± 20 | 558.4 ± 53.0 | 134 ± 13 |
|  | 4 | 36.5 ± 1.4 | 15.6 ± 0.4 | 242 ± 34 | 717.0 ± 63.9 | 179 ± 17 |

Note: DBH is the arithmetic mean of the diameter at breast height, BA is the basal area, and SDI is the stand density index.
