# Supplementary material for: Mountain Pine Beetle Dynamics and Reproductive Success in Post-Fire Lodgepole and Ponderosa Pine Forests in Northeastern Utah
Source: PLoS One. 2016 Oct 26;11(10):e0164738. doi: 10.1371/journal.pone.0164738 (PMC5082653; doi:10.1371/journal.pone.0164738)
Supplement: S2 Table — (DOCX) [file pone.0164738.s003.docx]

**S2 Table. Within-tree correlations between fire injury measures for lodgepole pine and ponderosa pine.**

| **Tree Species** | **Fire Injury Metric** |  | **TCD** | | |  | **CVS** | | |  | **CVC** | | |  | **CKR** | | |  | **BSP** | | |
| --- | --- | --- | --- | --- | --- | --- | --- | --- | --- | --- | --- | --- | --- | --- | --- | --- | --- | --- | --- | --- | --- |
|  |  |  | *R* | *R_s_* | *P* |  | *R* | *R_s_* | *P* |  | *R* | *R_s_* | *P* |  | *R* | *R_s_* | *P* |  | *R* | *R_s_* | *P* |
| Lodgepole Pine | BCI |  | 0.581 | 0.60 | ≤0.001 |  | 0.469 | 0.50 | ≤0.001 |  | 0.348 | 0.39 | ≤0.001 |  | 0.873 | 0.88 | ≤0.001 |  | 0.602 | 0.92 | ≤0.001 |
|  | BSP |  | 0.576 | 0.70 | ≤0.001 |  | 0.274 | 0.58 | ≤0.001 |  | 0.764 | 0.50 | ≤0.001 |  | 0.504 | 0.87 | ≤0.001 |  |  |  |  |
|  | CKR |  | 0.656 | 0.67 | ≤0.001 |  | 0.581 | 0.60 | ≤0.001 |  | 0.281 | 0.37 | ≤0.001 |  |  |  |  |  |  |  |  |
|  | CVC |  | 0.386 | 0.48 | ≤0.001 |  | 0.045 | 0.19 | ≤0.001 |  |  |  |  |  |  |  |  |  |  |  |  |
|  | CVS |  | 0.906 | 0.92 | ≤0.001 |  |  |  |  |  |  |  |  |  |  |  |  |  |  |  |  |
| Ponderosa Pine | BCI |  | 0.453 | 0.36 | ≤0.001 |  | 0.167 | 0.06 | 0.164 |  | 0.286 | 0.31 | ≤0.001 |  | 0.514 | 0.53 | ≤0.001 |  | 0.445 | 0.45 | ≤0.001 |
|  | BSP |  | 0.727 | 0.83 | ≤0.001 |  | 0.164 | 0.00 | 0.952 |  | 0.869 | 0.78 | ≤0.001 |  | 0.482 | 0.52 | ≤0.001 |  |  |  |  |
|  | CKR |  | 0.489 | 0.54 | ≤0.001 |  | 0.118 | 0.11 | 0.008 |  | 0.369 | 0.41 | ≤0.001 |  |  |  |  |  |  |  |  |
|  | CVC |  | 0.541 | 0.67 | ≤0.001 |  | -0.495 | -0.36 | ≤0.001 |  |  |  |  |  |  |  |  |  |  |  |  |
|  | CVS |  | 0.464 | 0.26 | ≤0.001 |  |  |  |  |  |  |  |  |  |  |  |  |  |  |  |  |

Note: *R* is the correlation coefficient, *R_s_* is the Spearman rank correlation coefficient, and the *P*-value for *R_s_* is displayed. See Table 1 for description of fire injury measures.
