# Supplementary material for: Mountain Pine Beetle Dynamics and Reproductive Success in Post-Fire Lodgepole and Ponderosa Pine Forests in Northeastern Utah
Source: PLoS One. 2016 Oct 26;11(10):e0164738. doi: 10.1371/journal.pone.0164738 (PMC5082653; doi:10.1371/journal.pone.0164738)
Supplement: S3 Table — (DOCX) [file pone.0164738.s004.docx]

**S3 Table. Model parameters for the number of mountain pine beetle, *Ips* spp., and other bark beetles caught in flight intercept traps in lodgepole and ponderosa pines each year.** Standard error, *P*-value from *z*-tests, likelihood ratio chi-square test and its associated *P*-value are displayed for each covariate.

| **Tree Species** | **Insect Species** | **Covariate** | **Coefficient** | **Std. Err.** | ***P_z_*** | **LRT** | ***P_LRT_*** |
| --- | --- | --- | --- | --- | --- | --- | --- |
| Lodgepole pine | Mountain pine beetle | Intercept (2007) | 2.823 | 0.111 | <0.001 | - | - |
|  |  | Year | - | - | - | 182.950 | <0.001 |
|  |  | 2009 | -0.046 | 0.085 | 0.583 | - | - |
|  |  | 2010 | -1.501 | 0.138 | <0.001 | - | - |
|  | *Ips* spp. | Intercept (2007) | 2.150 | 0.118 | <0.001 | - | - |
|  |  | Year | - | - | - | 58.649 | <0.001 |
|  |  | 2009 | -1.148 | 0.170 | <0.001 | - | - |
|  |  | 2010 | -1.714 | 0.145 | <0.001 | - | - |
|  | Other Scolytinae | Intercept (2007) | 3.395 | 0.124 | <0.001 | - | - |
|  |  | Year | - | - | - | 38.221 | <0.001 |
|  |  | 2009 | -0.233 | 0.065 | <0.001 | - | - |
|  |  | 2010 | 0.152 | 0.059 | <0.001 | - | - |
| Ponderosa pine | Mountain pine beetle | Intercept (2007) | 3.221 | 0.189 | <0.001 | - | - |
|  |  | Year | - | - | - | 393.410 | <0.001 |
|  |  | 2009 | -0.759 | 0.078 | <0.001 | - | - |
|  |  | 2010 | -2.010 | 0.127 | <0.001 | - | - |
|  | *Ips* spp. | Intercept (2007) | 1.479 | 0.279 | <0.001 | - | - |
|  |  | Year | - | - | - | 70.713 | <0.001 |
|  |  | 2009 | -1.555 | 0.231 | <0.001 | - | - |
|  |  | 2010 | -1.107 | 0.194 | <0.001 | - | - |
|  | Other Scolytinae | Intercept (2007) | 1.759 | 0.131 | <0.001 | - | - |
|  |  | Year | - | - | - | 255.100 | <0.001 |
|  |  | 2009 | 1.575 | 0.116 | <0.001 | - | - |
|  |  | 2010 | 1.290 | 0.119 | <0.001 | - | - |
