# Supplementary material for: Mountain Pine Beetle Dynamics and Reproductive Success in Post-Fire Lodgepole and Ponderosa Pine Forests in Northeastern Utah
Source: PLoS One. 2016 Oct 26;11(10):e0164738. doi: 10.1371/journal.pone.0164738 (PMC5082653; doi:10.1371/journal.pone.0164738)
Supplement: S4 Table — (DOCX) [file pone.0164738.s005.docx]

**S4 Table. Model parameters for binomial models of fire injury categories predicting tree mortality and MPB attack.**

|  |  |  | **Lodgepole** | | | | **Ponderosa** | | | |
| --- | --- | --- | --- | --- | --- | --- | --- | --- | --- | --- |
|  |  |  | **Estimate** | **Std. Error** | **z** | **P-value** | **Estimate** | **Std. Error** | **z** | **P-value** |
| MPB attack | TCD | intercept | -0.165 | 0.062 | -2.683 | 0.007 | 0.200 | 0.938 | 2.133 | 0.033 |
|  |  | TCD | -19.947 | 2.181 | -9.144 | < 0.001 | 22.112 | 2.566 | 8.619 | < 0.001 |
|  |  | TCD^2^ | -7.902 | 2.061 | -3.834 | < 0.001 | -13.601 | 2.478 | -5.489 | < 0.001 |
|  | CSV | intercept | -0.153 | 0.060 | -2.548 | 0.011 | 0.333 | 0.096 | 3.457 | < 0.001 |
|  |  | CSV | -15.259 | 2.141 | -7.127 | < 0.001 | 26.526 | 2.553 | 10.388 | < 0.001 |
|  |  | CSV^2^ | -3.672 | 2.042 | -1.799 | 0.072 | -2.185 | 2.386 | -0.916 | 0.360 |
|  | CVC | intercept | -0.182 | 0.063 | -2.893 | 0.004 | 0.294 | 0.087 | 3.369 | < 0.001 |
|  |  | CVC | -19.202 | 4.169 | -4.606 | < 0.001 | -3.148 | 2.082 | -1.512 | 0.131 |
|  |  | CVC^2^ | -2.784 | 2.956 | -0.942 | 0.346 | -15.356 | 2.574 | -5.965 | < 0.001 |
|  | CKR | intercept (0) | 0.122 | 0.097 | 1.259 | 0.208 | -0.586 | 0.170 | -3.450 | < 0.001 |
|  |  | 1 | 0.571 | 0.284 | 2.008 | 0.045 | 1.013 | 0.256 | 3.957 | < 0.001 |
|  |  | 2 | 0.437 | 0.242 | 1.808 | 0.071 | 1.080 | 0.247 | 4.374 | < 0.001 |
|  |  | 3 | 0.621 | 0.246 | 2.530 | 0.011 | 1.567 | 0.294 | 5.338 | < 0.001 |
|  |  | 4 | -0.884 | 0.136 | -6.496 | < 0.001 | 0.977 | 0.256 | 3.823 | < 0.001 |
|  | BCP | intercept | -0.200 | 0.064 | -3.106 | 0.002 | 0.297 | 0.095 | 3.129 | 0.002 |
|  |  | BCP | -24.573 | 3.617 | -6.794 | < 0.001 | 8.783 | 2.239 | 3.922 | < 0.001 |
|  |  | BCP^2^ | 1.882 | 2.762 | 0.681 | 0.496 | -28.538 | 2.767 | -10.313 | < 0.001 |
|  | BCR | intercept (0) | 0.204 | 0.087 | 2.337 | 0.019 | -2.169 | 0.528 | -4.110 | < 0.001 |
|  |  | 1 | 0.169 | 0.160 | 1.053 | 0.292 | 1.681 | 0.582 | 2.890 | 0.004 |
|  |  | 2 | -0.813 | 0.152 | -5.336 | < 0.001 | 2.750 | 0.537 | 5.118 | < 0.001 |
|  |  | 3 | -1.987 | 0.263 | -7.559 | < 0.001 | 2.204 | 0.591 | 3.732 | < 0.001 |
| Tree Mortality | TCD | intercept | 1.520 | 0.211 | 7.196 | < 0.001 | 0.044 | 0.127 | 0.344 | 0.731 |
|  |  | TCD | 87.520 | 9.515 | 9.199 | < 0.001 | 47.634 | 3.481 | 13.686 | < 0.001 |
|  |  | TCD^2^ | 10.852 | 3.348 | 3.241 | 0.001 | 14.155 | 2.966 | 4.772 | < 0.001 |
|  | CSV | intercept | 1.290 | 0.178 | 7.256 | < 0.001 | 0.084 | 0.087 | 0.963 | 0.335 |
|  |  | CSV | 78.002 | 8.993 | 8.674 | < 0.001 | 8.289 | 2.223 | 3.728 | < 0.001 |
|  |  | CSV^2^ | 6.474 | 3.878 | 1.669 | 0.095 | 15.452 | 2.235 | 6.913 | < 0.001 |
|  | CVC | intercept | 0.769 | 0.129 | 5.945 | < 0.001 | 0.730 | 0.271 | 2.692 | 0.007 |
|  |  | CVC | 45.893 | 15.562 | 2.949 | 0.003 | 58.912 | 12.245 | 4.811 | < 0.001 |
|  |  | CVC^2^ | -18.427 | 7.940 | -2.321 | 0.020 | -2.774 | 4.484 | -0.619 | 0.536 |
|  | CKR | intercept (0) | -1.169 | 0.114 | -10.259 | < 0.001 | -1.570 | 0.216 | -7.285 | < 0.001 |
|  |  | 1 | 0.615 | 0.285 | 2.156 | 0.031 | 1.359 | 0.286 | 4.747 | < 0.001 |
|  |  | 2 | 1.123 | 0.242 | 4.646 | < 0.001 | 1.388 | 0.278 | 5.001 | < 0.001 |
|  |  | 3 | 2.016 | 0.257 | 7.854 | < 0.001 | 2.669 | 0.327 | 8.156 | < 0.001 |
|  |  | 4 | 4.886 | 0.314 | 15.582 | < 0.001 | 4.027 | 0.409 | 9.851 | < 0.001 |
|  | BCP | intercept | 1.876 | 0.306 | 6.125 | < 0.001 | 0.439 | 0.193 | 2.273 | 0.023 |
|  |  | BCP | 93.011 | 27.745 | 3.352 | < 0.001 | 59.491 | 6.407 | 9.286 | < 0.001 |
|  |  | BCP^2^ | -70.664 | 13.120 | -5.386 | < 0.001 | -4.503 | 4.207 | -1.070 | 0.284 |
|  | BCR | intercept (0) | -0.773 | 0.093 | -8.290 | < 0.001 | -2.485 | 0.601 | -4.135 | < 0.001 |
|  |  | 1 | 1.671 | 0.173 | 9.644 | < 0.001 | 0.555 | 0.699 | 0.794 | 0.427 |
|  |  | 2 | 3.397 | 0.255 | 13.309 | < 0.001 | 2.831 | 0.609 | 4.650 | < 0.001 |
|  |  | 3 | 18.339 | 344.341 | 0.053 | 0.958 | 3.607 | 0.675 | 5.343 | < 0.001 |
