# Supplementary material for: Mountain Pine Beetle Dynamics and Reproductive Success in Post-Fire Lodgepole and Ponderosa Pine Forests in Northeastern Utah
Source: PLoS One. 2016 Oct 26;11(10):e0164738. doi: 10.1371/journal.pone.0164738 (PMC5082653; doi:10.1371/journal.pone.0164738)
Supplement: S5 Table — (DOCX) [file pone.0164738.s006.docx]

**S5 Table. Likelihood ratio tests for binomial models of fire injury categories predicting tree mortality and MPB attack.**

|  |  | **Lodgepole** | |  | **Ponderosa** | |  |
| --- | --- | --- | --- | --- | --- | --- | --- |
|  |  | **LR Chi Sq** | **DF** | **pr(>\|z\|)** | **LR Chi Sq** | **DF** | **pr(>\|z\|)** |
| MPB attack | TCD | 103.86 | 2 | < 0.001 | 122 | 2 | < 0.001 |
|  | CSV | 56.61 | 2 | < 0.001 | 141.41 | 2 | < 0.001 |
|  | CVC | 46.42 | 2 | < 0.001 | 47.91 | 2 | < 0.001 |
|  | CKR | 89.85 | 4 | < 0.001 | 39.06 | 4 | < 0.001 |
|  | BCP | 95.91 | 2 | < 0.001 | 154.34 | 2 | < 0.001 |
|  | BCR | 106.77 | 3 | < 0.001 | 57.91 | 3 | < 0.001 |
| Tree Mortality | TCD | 514.99 | 2 | < 0.001 | 371.88 | 2 | < 0.001 |
|  | CSV | 431.14 | 2 | < 0.001 | 64.63 | 2 | < 0.001 |
|  | CVC | 110.79 | 2 | < 0.001 | 243.89 | 2 | < 0.001 |
|  | CKR | 666.3 | 4 | < 0.001 | 190.58 | 4 | < 0.001 |
|  | BCP | 508.41 | 2 | < 0.001 | 352.01 | 2 | < 0.001 |
|  | BCR | 484.06 | 3 | < 0.001 | 105.08 | 3 | < 0.001 |
