# Supplementary material for: Mountain Pine Beetle Dynamics and Reproductive Success in Post-Fire Lodgepole and Ponderosa Pine Forests in Northeastern Utah
Source: PLoS One. 2016 Oct 26;11(10):e0164738. doi: 10.1371/journal.pone.0164738 (PMC5082653; doi:10.1371/journal.pone.0164738)
Supplement: S6 Table — (DOCX) [file pone.0164738.s007.docx]

**S6 Table. Parameters providing the best fit for the likelihood of attack by mountain pine beetle on lodgepole pine.**  Generalized linear mixed models using fire injury measures, tree mensuration characteristics and year of attack. Standard error, *P*-value from z-tests, likelihood ratio chi-square test and its associated p-value are displayed for each covariate.

| **Species** | **Model** | **Covariate** | **Coefficient** | **Std. Err.** | **P_z_** | **LRT** | **P_LRT_** |
| --- | --- | --- | --- | --- | --- | --- | --- |
| Lodgepole  pine | Mass | Intercept (2007) | -8.133 | 0.906 | <0.001 | - | - |
|  |  | CVS | 0.02 | 0.003 | <0.001 | 38.033 | <0.001 |
|  |  | CKR | 1.112 | 0.235 | <0.001 | 26.388 | <0.001 |
|  |  | BCI | -0.58 | 0.163 | <0.001 | 12.492 | <0.001 |
|  |  | DBH | 0.053 | 0.012 | <0.001 | 32.43 | <0.001 |
|  |  | Year | - | - | - | 43.302 | <0.001 |
|  |  | 2008 | 1.43 | 0.961 | 0.137 | - | - |
|  |  | 2009 | 3.541 | 0.85 | <0.001 | - | - |
|  |  | 2010 | 3.651 | 0.862 | <0.001 | - | - |
|  |  | CKR*Year | - | - | - | 23.168 | <0.001 |
|  |  | CKR*2008 | -0.406 | 0.263 | 0.123 | - | - |
|  |  | CKR*2009 | -0.742 | 0.259 | 0.013 | - | - |
|  |  | CKR*2010 | -1.422 | 0.444 | 0.003 | - | - |
|  | Strip | Intercept (2007) | -5.751 | 0.37 | <0.001 |  |  |
|  |  | TCD | -0.009 | 0.003 | 0.003 | 9.504 | 0.002 |
|  |  | CKR | 0.674 | 0.09 | <0.001 | 23.783 | <0.001 |
|  |  | BSP | -0.032 | 0.01 | 0.002 | 15.205 | <0.001 |
|  |  | DBH | 0.075 | 0.008 | <0.001 | 100.28 | <0.001 |
|  |  | Year | - | - | - | 67.294 | <0.001 |
|  |  | 2008 | 2.562 | 0.287 | <0.001 | - | - |
|  |  | 2009 | 0.831 | 0.33 | 0.012 | - | - |
|  |  | 2010 | 1.339 | 0.332 | <0.001 | - | - |
|  |  | CKR*Year | - | - | - | 79.832 | <0.001 |
|  |  | CKR*2008 | -0.788 | 0.103 | <0.001 | - | - |
|  |  | CKR*2009 | -0.334 | 0.143 | 0.019 | - | - |
|  |  | CKR*2010 | -0.079 | 0.147 | 0.591 | - | - |
