# Supplementary material for: Mountain Pine Beetle Dynamics and Reproductive Success in Post-Fire Lodgepole and Ponderosa Pine Forests in Northeastern Utah
Source: PLoS One. 2016 Oct 26;11(10):e0164738. doi: 10.1371/journal.pone.0164738 (PMC5082653; doi:10.1371/journal.pone.0164738)
Supplement: S7 Table — (DOCX) [file pone.0164738.s008.docx]

**S7 Table. Parameters providing the best fit for the likelihood of attack by mountain pine beetle on ponderosa pine.**  Generalized linear mixed models using fire injury measures, tree mensuration characteristics and year of attack. Standard error, *P*-value from *z*-tests, likelihood ratio chi-square test (LRT) and its associated *P*-value are displayed for each covariate.

| **Species** | **Model** | **Covariate** | **Coefficient** | **Std. Err.** | ***P*_z_** | **LRT** | ***P*_LRT_** |
| --- | --- | --- | --- | --- | --- | --- | --- |
| Ponderosa  pine | Mass | Intercept (2007) | -7.526 | 0.683 | <0.001 | - | - |
|  |  | TCD | 0.047 | 0.006 | <0.001 | 98.11 | <0.001 |
|  |  | CKR | 0.323 | 0.086 | <0.001 | 14.509 | <0.001 |
|  |  | DBH | 0.028 | 0.007 | <0.001 | 16.861 | <0.001 |
|  |  | Year | - | - | - | 10.409 | 0.015 |
|  |  | 2008 | -0.564 | 0.302 | 0.062 | - | - |
|  |  | 2009 | 0.458 | 0.291 | 0.116 | - | - |
|  |  | 2010 | -0.417 | 0.425 | 0.327 | - | - |
|  | Strip | Intercept (2007) | -4.183 | 0.398 | <0.001 | - | - |
|  |  | CVS | 0.023 | 0.004 | <0.001 | 37.481 | <0.001 |
|  |  | DBH | 0.027 | 0.006 | <0.001 | 18.705 | <0.001 |
|  |  | Year | - | - | - | 40.346 | <0.001 |
|  |  | 2008 | -0.796 | 0.226 | <0.001 | - | - |
|  |  | 2009 | -1.2 | 0.286 | <0.001 | - | - |
|  |  | 2010 | -1.722 | 0.397 | <0.001 | - | - |
