# Supplementary material for: Mountain Pine Beetle Dynamics and Reproductive Success in Post-Fire Lodgepole and Ponderosa Pine Forests in Northeastern Utah
Source: PLoS One. 2016 Oct 26;11(10):e0164738. doi: 10.1371/journal.pone.0164738 (PMC5082653; doi:10.1371/journal.pone.0164738)
Supplement: S8 Table — (DOCX) [file pone.0164738.s009.docx]

**S8 Table. Parameters for mountain pine beetle reproduction models.**  Includes the number of emerging mountain pine beetles, ovipositional galleries, and ratio of emerging beetles to ovipositional galleries for caged lodgepole and ponderosa pines with mountain pine beetle attack using generalized linear mixed models. Standard error, *P*-value from *z*-tests, likelihood ratio chi-square test (LRT) and its associated *P*-value are displayed for each covariate.

| **Tree Species** | **Model** | **Covariate** | **Coefficient** | **Std. Err.** | **P_z or t_** | **LRT** | **P_LRT_** |
| --- | --- | --- | --- | --- | --- | --- | --- |
| Lodgepole pine | Emerging beetles | Intercept (2007) | 3.542 | 0.246 | <0.001 | - | - |
|  |  | Cage Burn | -0.165 | 0.061 | 0.007 | 7.347 | 0.007 |
|  |  | Ips spp. | 0.039 | 0.006 | <0.001 | 60.497 | <0.001 |
|  |  | Other beetles | -0.165 | 0.005 | <0.001 | 145.000 | <0.001 |
|  |  | Year | - | - | - | 15.931 | <0.001 |
|  |  | 2009 | 0.802 | 0.317 | 0.012 | - | - |
|  |  | 2010 | -0.371 | 0.317 | 0.242 | - | - |
|  | Ovipositional galleries | Intercept (2007) | 3.374 | 0.057 | <0.001 | - | - |
|  |  | Cage burn | -0.572 | 0.056 | <0.001 | 96.352 | <0.001 |
|  |  | Other Beetles | -0.017 | 0.005 | <0.001 | 13.187 | <0.001 |
|  |  | Year | - | - | - | 6.617 | 0.037 |
|  |  | 2009 | 0.037 | 0.068 | 0.592 | - | - |
|  |  | 2010 | 0.158 | 0.068 | 0.02 | - | - |
|  | Ratio of  Emerging beetles ovipositional galleries | Intercept (2007) | 2.813 | 0.895 | 0.002 | - | - |
|  |  | Year | - | - | - | 13.388 | 0.001 |
|  |  | 2009 | 1.423 | 1.164 | 0.225 | - | - |
|  |  | 2010 | -1.854 | 1.156 | 0.113 | - | - |
| Ponderosa pine | Emerging beetles | Intercept (2007) | 3.803 | 0.509 | <0.001 | - | - |
|  |  | BCI | -0.396 | 0.188 | 0.036 | 4.219 | 0.04 |
|  |  | Cage burn | 0.692 | 0.132 | <0.001 | 28.964 | <0.001 |
|  |  | Ips spp. | 0.041 | 0.005 | <0.001 | 80.852 | <0.001 |
|  |  | Other beetles | -0.01 | 0.004 | 0.01 | 6.823 | 0.009 |
|  |  | Year | - | - | - | 34.788 | <0.001 |
|  |  | 2009 | -1.521 | 0.398 | <0.001 | - | - |
|  |  | 2010 | -2.677 | 0.409 | <0.001 | - | - |
|  | Ovipositional galleries | Intercept (2007) | 3.316 | 0.064 | <0.001 |  |  |
|  |  | BSP | -0.014 | 0.002 | <0.001 | 35.324 | <0.001 |
|  |  | Cage burn | -0.224 | 0.075 | 0.003 | 8.51 | 0.003 |
|  |  | Other beetles | -0.008 | 0.002 | <0.001 | 21.706 | <0.001 |
|  | Ratio of  Emerging beetles ovipositional galleries | Intercept (2007) | 2.813 | 0.355 | <0.001 | - | - |
|  |  | Year | - | - | - | 28.613 | <0.001 |
|  |  | 2009 | -1.987 | 0.459 | <0.001 | - | - |
|  |  | 2010 | -2.569 | 0.453 | <0.001 | - | - |
