# Supplementary material for: Mountain Pine Beetle Dynamics and Reproductive Success in Post-Fire Lodgepole and Ponderosa Pine Forests in Northeastern Utah
Source: PLoS One. 2016 Oct 26;11(10):e0164738. doi: 10.1371/journal.pone.0164738 (PMC5082653; doi:10.1371/journal.pone.0164738)
Supplement: S9 Table — (DOCX) [file pone.0164738.s010.docx]

**S9 Table. Mountain pine beetle attacks and emergence in fire injured and uninjured lodgepole and ponderosa pines with and without *Ips* spp.**

| **Species** | **Category** | | **Attacked Trees (*n*)** | **Mass Attack (%)** | **Strip Attack (%)** | **Failed Attack (%)** | **Emergence ± SE (*n* cages)** |
| --- | --- | --- | --- | --- | --- | --- | --- |
| Lodgepole Pine | Fire Injured | |  |  |  |  |  |
|  |  | MPB | 249 | 37.7 a | 38.2 a | 24.1 a | 51.4 ± 7.2 (75) a |
|  |  | MPB + *Ips* spp. | 138 | 16.0 b | 57.2 b | 26.8 a | 68.4 ± 8.8 (55) b |
|  |  |  |  |  |  |  |  |
|  | Uninjured | |  |  |  |  |  |
|  |  | MPB | 133 | 34.6 a | 24.8 c | 40.6 b | 49.7 ± 13.3 (20) a |
|  |  | MPB + *Ips* spp. | 26 | 50.0 a | 46.2 abc | 3.8 a | 72.1 ± 27.5 (8) b |
| Ponderosa Pine | Fire Injured | |  |  |  |  |  |
|  |  | MPB | 316 | 39.2 a | 33.5 a | 27.2 a | 16.4 ± 2.9 (109) a |
|  |  | MPB + *Ips* spp. | 21 | 52.4 a | 19.0 a | 28.6 a | 45.4 ± 9.2 (31) b |
|  |  |  |  |  |  |  |  |
|  | Uninjured | |  |  |  |  |  |
|  |  | MPB | 0 | - | - | - | 8.7 ± 2.1 (24) c |
|  |  | MPB + *Ips* spp. | 0 | - | - | - | - |

Note: Presence of *Ips* spp. was based on post-fire infestation that occurred prior to or concurrent with mountain pine beetle attack. Values within a column with differing letters for the same tree species, show statistical difference (*α* ≤ 0.05) based on Tukey HSD contrasts from mixed models. Uninjured ponderosa pines were located within the study area, but not within plots.
